# Variation in body size and weight status among Hindu and Muslim Indian males born in the 1890s through the 1950s

**DOI:** 10.1038/s41598-024-54637-1

**Published:** 2024-02-20

**Authors:** Grażyna Liczbińska, Rajesh K. Gautam, Premananda Bharati, Robert M. Malina

**Affiliations:** 1 Institute of Human Biology and Evolution, Faculty of Biology, Adam Mickiewicz University, Poznań, Poland; 2 Department of Anthropology, Dr. Harisingh Gour Vishwavidyalaya (A Central University), Sagar, Madhya Pradesh India; 3 Biological Anthropology Unit, Indian Statistical Institute, Kolkata, India; 4 Professor Emeritus, Department of Kinesiology and Health Education, University of Texas, Austin, TX 78705 USA; 5 Adjunct Professor, School of Public Health and Information Sciences, University of Louisville, Louisville, KY USA

**Keywords:** BMI, Height, Weight status, Religion, Nutrition, Social roles, Developmental biology, Ecology

## Abstract

Hindus and Muslims represent the two largest religions in India, and also differ in nutritional status, health-related habits and standard of living associated with economic disparities. In this context, the present study considered estimated secular changes in body size, proportions, and weight status among Hindu and Muslim Indian men. The data are from anthropological surveys in the 1970s which included measurements of height, weight and sitting height of 43,950 males 18–84 years (birth years 1891–1957). Leg length was estimated; the BMI and sitting height/height ratio were calculated. Heights of men 35 + years were adjusted for estimated height loss with age. Weight status was also classified relative to WHO criteria for the BMI. Anthropometric characteristics of the two groups were compared with MANCOVA with age and geographic region as covariates. Linear regression of height on year of birth was also used to estimate secular change in each group. Heights, weights, and BMIs tended to be, on average, greater among Muslim than Hindu men at most ages, while distributions by weight status between groups were negligible. Sitting height was greater among Muslim men but estimated leg length did not differ between groups; the sitting height/height ratio thus suggested proportionally shorter legs among Muslim men. Results of the regression analyses indicated negligible differences in secular change between groups across the total span of birth years but indicated a decline in adjusted heights of men in both groups born between 1891 through 1930s and little secular change among those born in the 1930s through 1957. The variation in heights, weights and BMIs between Muslim and Hindu men at most ages suggested variation in socio-economic status and dietary habits between the groups, whereas the negligible estimated secular changes in height between groups likely reflected economic, social, and nutritional conditions during the interval of British rule and the transition to independence.

## Introduction

Variation in height and weight, life expectancy, and infant and middle-age morbidity and mortality within and among populations likely reflect the biological standard of living which often defines the well-being of populations associated with economic development, higher living standards, and by inference nutritional conditions and health status^1–9^. Meanwhile, the literature also emphasizes a relationship among health status and related behaviors, demographic factors, and nutritional habits within and among religious denominations^10^. In addition to beliefs per se, religions are generally defined by specific norms and rules, which influence the way and style of life of individuals, families, and communities^10^. These behaviors also influence nutritional habits and health status, and of course, conditions into which children are born, grow and mature. Moreover, some research suggests that religious preference or denomination also influences quality of life and standard of living through effects on socioeconomic conditions and lifestyle as evident in observations in earlier societies^10,11^.

Hinduism and Islam are the two largest religions in India^12^. Their respective followers differ in lifestyle, tradition, nutritional habits, and economic conditions^13,14^, which may contribute to differences in body size, specifically height, an environmentally sensitive characteristic^15–17^. Meanwhile, social, and economic conditions and several natural disasters in India at the end of the 19th and first half of the twentieth century likely contributed to persistent marginal nutritional status and at times chronic undernutrition over time^18^. These conditions may have also influenced nutritional habits and economic conditions between Hindus and Muslims, and in turn, variation in body size between the groups. Previous research on Indian men 18–84 years has already shown a lack of significant secular changes in body size of men born during the interval of British rule in India and during the early years after independence in late 1947^18^, and variation in body size associated with socio-economic disparities and ecological differences among regions^19^.

The purpose of the present study is twofold. It initially compares the body size, proportions, and weight status of a national sample of Indian males surveyed in the 1970s who were classified by religious preference as Hindu and Muslim, and then compares estimated secular changes in heights across the interval of birth years represented in the respective samples. The birth years spanned 1891 to 1957, an interval that included several important events in Indian history during the second half of the nineteenth century through the 1950s. The interval was dominated by British rule (1858–1947), while the Bengal famine (1940–1943) and several other famines, stresses associated with World War II, the immediate post-war years, and the initial years of independence (late 1947 onward) were additional factors. The men comprising the study sample were conceived, developed prenatally, and subsequently grew and matured from infancy into young adulthood during this interval.

Research addressing variation in biological characteristics associated with the standard of living in India under British rule and during the early years of independence is apparently not available. The present study provides an insight into the standard of living and its impact on the physical growth of the population under colonial rule and thus complements the economic and social history of India during this interval. The study also contributes to our understanding of the biological welfare of populations during an interval lacking traditional measures of biological and economic well-being.

## Results

**Figure 1:**
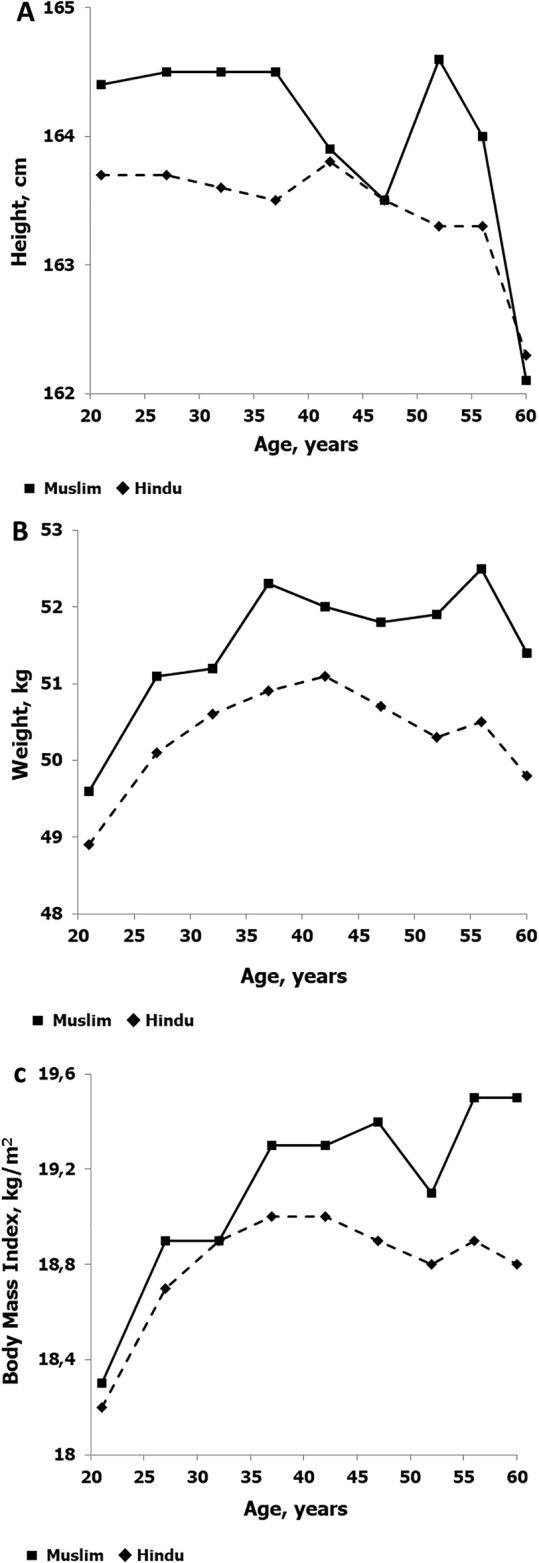
Mean heights (**A**), weights (**B**) and BMIs (**C**) in Hindus and Muslims by age groups.

Descriptive statistics by chronological age groups are summarized in Table 1 for age, year of birth and anthropometric characteristics. The table also includes the corresponding statistics for heights adjusted for estimated height loss with age among men 35 + years. Trends in means for height, weight and BMI of Muslim and Hindu by age groups are illustrated in Fig. 1A–C. Except among men 40–44 years, mean observed heights among Muslim men are, on average, greater than observed heights among Hindu men (Fig. 1A). The differences are significant 18 through 39 years, while differences in subsequent age groups are not significant except among men 50–54 years (Table 1). The trend in adjusted heights, i.e., adjusted for estimated height loss with age, is similar to that for measured heights among men 35 + years of age (Table 1). In contrast, mean weights are significantly heavier among Muslim men in all age groups except 60 + years. Mean weights increase with age from 18 years through the early 40 s in both Muslim and Hindu men, and then decline with age among Hindu men but are variable among Muslim men through the late 50 s and then decline (Fig. 1B). Although the differences are relatively small, mean BMIs are significantly higher among Muslim than Hindu men in all age groups except 30–34 years and 50–54 years (Table 1). Mean BMIs (Fig. 1C) show a pattern of change with age in both Muslim and Hindu men that is similar to that noted for body weight.

**Table 1:** Sample sizes, means (M) and standard deviations (SD) for year of birth, age, and several anthropometric characteristics, including adjusted heights for individuals 35 years and older, of Hindu (H) and Muslim (M) men by age groups.

Age group	Religion	N	Year of birth		Age, years		Height, cm		Weight, kg		BMI, kg/m^2^		Sitting Ht, cm		Est Leg, Lt, cm		Sit Ht ratio		Adjusted Ht, cm	
			M	SD	M	SD	M	SD	M	SD	M	SD	M	SD	M	SD	M	SD	M	SD
18–24	H	8101	1954	2.0	21.1	1.9	163.7	6.1	48.9	6.2	18.2	1.8	83.6	3.8	80.1	4.5	51.1	1.7		
	M	1200	1954	1.9	21.2	1.9	164.4	6.0	49.6	6.3	18.3	2.0	84.2	3.9	80.2	4.5*	51.2	1.6		
25–29	H	7199	1948	1.4	26.8	1.4	163.7	6.3	50.1	7.0	18.7	2.1	83.3	3.8	80.3	4.8	50.9	1.8		
	M	1005	1948	1.5	26.6	1.5	164.5	6.1	51.1	7.6	18.9	2.4	84.1	3.8	80.4	4.5*	51.1	1.7		
30–34	H	5791	1944	1.4	31.5	1.4	163.6	6.3	50.6	7.7	18.9	2.4	83.3	3.8	80.3	4.9	50.9	1.8		
	M	715	1944	1.4	31.4	1.4	164.5	6.0	51.2	8.0	18.9	2.6*	83.9	3.8	80.5	4.6*	51.0	1.8*		
35–39	H	5099	1939	1.5	36.5	1.5	163.5	6.4	50.9	8.1	19.0	2.5	83.0	3.8	80.5	4.9	50.8	1.8	163.6	6.4
	M	679	1939	1.5	36.4	1.5	164.5	6.2	52.3	8.7	19.3	2.8	83.9	3.7	80.6	4.7*	51.0	1.8	164.6	6.2
40–44	H	4243	1934	1.3	41.3	1.3	163.8	6.4	51.1	8.4	19.0	2.6	83.0	3.9	80.8	5.1	50.7	1.9	164.1	6.4
	M	503	1934	1.3	41.2	1.3	163.9	6.1*	52.0	8.8	19.3	2.9	83.6	3.3	80.4	4.5*	51.0	1.6	164.2	6.1*
45–49	H	3270	1929	1.5	46.4	1.5	163.5	6.3	50.7	8.4	18.9	2.7	83.0	3.9	80.5	4.8	50.8	1.9	164.0	6.3
	M	404	1929	1.4	46.1	1.4	163.5	6.1*	51.8	8.7	19.4	2.9	83.4	3.6	80.1	4.8*	51.0	1.8	164.0	6.1*
50–54	H	3061	1924	1.4	51.2	1.4	163.3	6.5	50.3	8.4	18.8	2.7	82.8	3.9	80.5	5.0	50.7	1.9	164.2	6.5
	M	352	1924	1.3	51.2	1.3	164.6	6.4	51.9	9.1	19.1	3.0*	83.4	3.7	81.1	4.8	50.7	1.8*	165.5	6.4
55–59	H	1480	1919	1.3	55.9	1.3	163.3	6.5	50.5	8.9	18.9	2.9	82.5	3.8	80.8	5.1	50.5	1.9	164.7	6.5
	M	183	1919	1.3	56.0	1.3	164.0	5.7*	52.5	9.7	19.5	3.3	83.4	3.8	80.5	4.2*	50.9	1.7	165.4	5.7*
60 +	H	532	1913	3.3	60.5	1.0	162.3	6.6	49.8	9.5	18.8	3.0	81.6	3.8	80.6	5.2	50.3	1.9	164.4	6.7
	M	98	1913	3.6	60.3	0.8	162.1	6.2*	51.4	9.2*	19.5	3.0	81.9	3.9*	80.2	4.4*	50.6	1.7*	164.3	6.1*

*Pairwise comparisons of anthropometric characteristics indicated with an asterisk (*) are not significant; all other pairwise differences are statistically significant (p < 0.05, p < 0.01 or p < 0.001) based on age-group specific MANCOVAs with age, age squared and geographic area as covariates.

**Figure 2:**
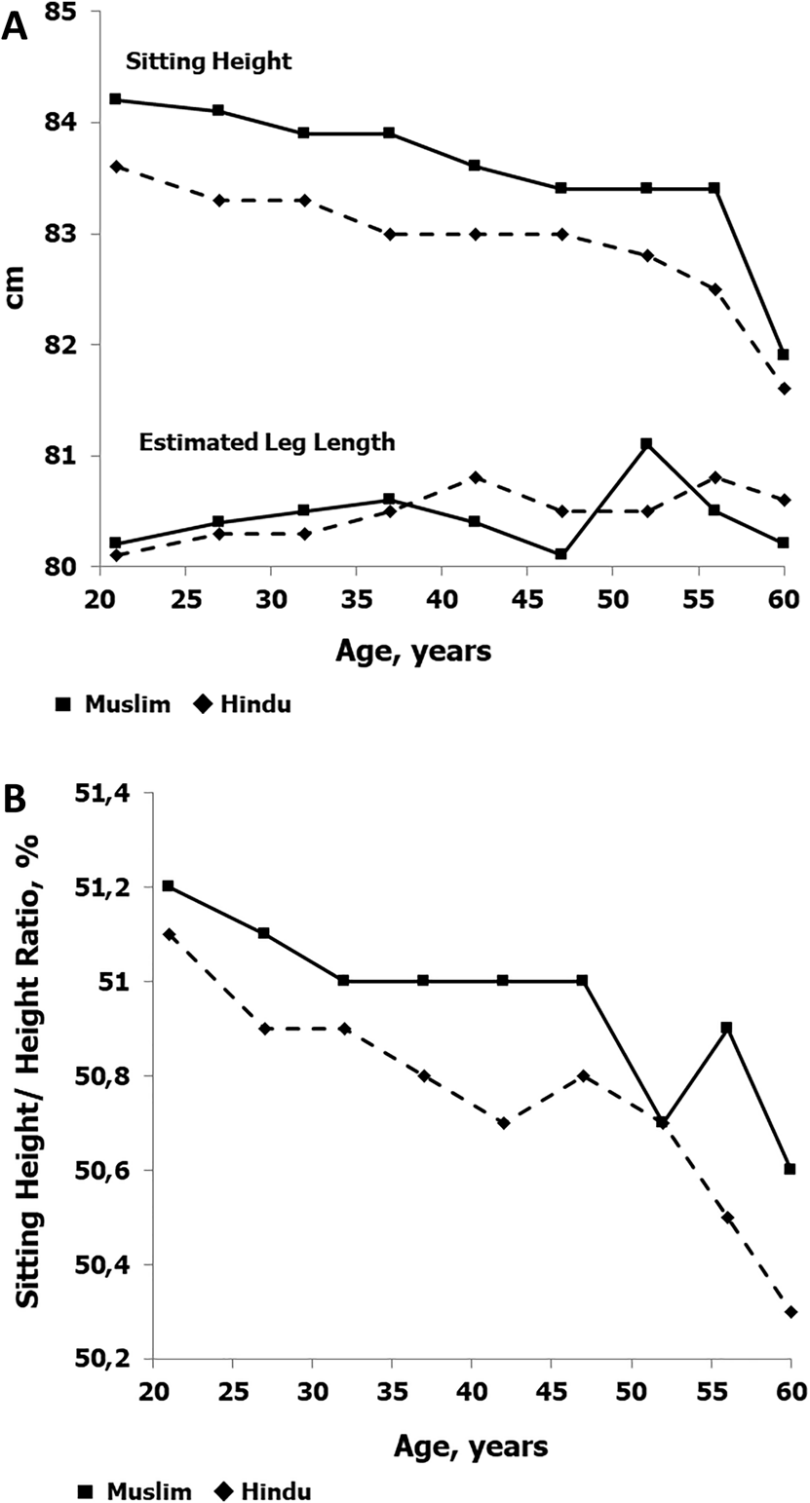
Sitting height and estimated leg lengths (**A**) and sitting height/height ratio (**B**) in Hindus and Muslims by age groups.

Corresponding trends in sitting height and estimated leg length are illustrated in Fig. 2A. Sitting height declines with age in both Muslim and Hindu men, while estimated leg length increases slightly between 18–24 years and 25–29 years, and then varies by age group. Note, leg length is estimated as the difference between height and sitting height and is influenced by the decline in sitting height with increasing age. Nevertheless, sitting height is significantly greater among Muslim men across the age range except at 60 + years, while estimated leg length does not systematically differ between Muslim and Hindu men (Table 1). The sitting height/height ratio (Fig. 2B) declines systematically across the age range in both Muslim and Hindu men, but is, on average, consistently higher among Muslim men; differences in the ratio are significant in all age groups except 30–34, 50–54 and 60 + years.

The distributions of Muslim and Hindu men by weight status within each age group are summarized in Table 2. The BMI cut-offs for levels of thinness in the two WHO criteria are identical. There are no consistent differences in the distributions of weight status among Muslim and Hindu men across the age range. About 60% of Muslim and Hindu men 18–24 years are classified as thin, while from 25–29 through 40–44 years about 50% of the Muslim and Hindu men are thin; subsequently, proportionally more Hindu than Muslim men are classified as thin. The prevalence of men classified as mildly or moderately thin is reasonably stable among both Muslim and Hindu men across the age range except in the oldest group, while the percentage of Muslim and Hindu men classified as severely thin is relatively stable from 18–24 to 45–49 years and increases with age through 60 + years in both groups.

**Table 2:** Weight status based on the BMI among Indian Hindu (H) and Muslim (M) men by age groups*

Age group	Religion	N	Thinness						Weight Status WHO Criteria						WHO Asia–Pacific Criteria							
			Severe		Moderate		Mild		Normal		Overweight		Obese		Normal		Overweight		Obese-1		Obese-2	
			(< 16.00)		(16.00–16.99)		(17.00–18.49)		(18.50–24.99)		(25.00–29.99)		(≥ 30.00)		(18.50–22.99)		(23.00–24.99)		(25.00–29.99)		(≥ 30.00)	
			n	%	n	%	n	%	n	%	n	%	n	%	n	%	n	%	n	%	n	%
18–24	H	8111	766	9	1274	16	2864	35	3181	39	25	0.3	1	0.01	3079	38	102	1	25	0.3	1	0.01
	M	1203	102	8	190	16	409	34	492	41	10	1	0	–	475	39	17	1	10	0.8	0	–
25–29	H	7199	518	7	992	14	2196	31	3397	47	93	1	3	0.04	3217	45	180	3	93	1	3	0.04
	M	1005	67	7	133	13	306	30	477	47	19	2	3	0.3	440	44	37	4	19	2	3	0.3
30–34	H	5791	435	8	718	12	1699	29	2804	48	126	2	9	0.2	2587	45	217	4	126	2	9	0.2
	M	715	66	9	91	13	197	28	338	47	20	3	3	0.4	308	43	30	4	20	3	3	0.4
35–39	H	5099	377	7	603	12	1470	29	2509	49	129	3	11	0.2	2273	45	236	5	129	3	11	0.2
	M	679	52	8	85	13	168	25	354	52	18	3	3	0.4	305	45	49	7	18	3	3	0.4
40–44	H	4243	382	9	510	12	1145	27	2073	49	119	3	14	0.3	1885	44	188	4	119	3	14	0.3
	M	503	41	8	56	11	133	26	255	51	13	3	5	1	227	45	28	6	13	3	5	1
45–49	H	3270	303	9	430	13	934	29	1498	46	100	3	5	0.2	1343	41	155	5	100	3	5	0.2
	M	404	38	9	46	11	85	21	215	53	18	4	2	0.5	190	47	25	6	18	4	2	0.5
50–54	H	3061	317	10	418	14	863	29	1372	45	85	3	6	0.2	1237	40	135	4	85	3	6	0.2
	M	352	49	14	43	12	64	18	180	51	16	5	0	–	160	45	20	6	16	5	0	–
55–59	H	1480	180	12	223	15	363	25	659	45	49	3	6	0.4	581	39	78	5	49	3	6	0.4
	M	183	24	13	18	10	35	19	91	50	14	8	1	0.5	80	44	11	6	14	8	1	0.5
60 +	H	532	76	14	73	14	126	24	229	43	27	5	1	0.2	206	39	23	4	27	5	1	0.2
	M	98	12	12	9	9	18	18	55	56	4	4	0	–	47	48	8	8	4	4	0	–

*Criteria for weight status are those of the World Health Organization^54,55,56^. The criteria for categories of thinness were not modified in the proposed criteria for Asia–Pacific populations.

Relative to the commonly used WHO criteria, the proportions of men classified as normal in weight status is similar among Muslim and Hindu men 18–24 through 30–34 years but beginning about 35–39 years proportionally more Muslim than Hindu men are classified as normal in weight status (Table 2). The trend is similar for normal weight status using the WHO Asia–Pacific criteria among Muslim and Hindu men 18–24 through 40–44 years; subsequently, more Muslim than Hindu men are classified as normal in weight status. Regardless of the WHO criteria used, the prevalence of overweight and obesity among Muslim and Hindu men is low, and the estimated difference in prevalence between groups is small.

Results of the three pairs of linear regressions of height and adjusted heights on year of birth among Hindu and Muslim men are summarized in Table 3. The first regression in each pair is based on measured heights, while the second is based on the measured heights of men 18 through 34 years and adjusted heights of men 35 + years of age.

**Table 3:** Regressions of measured heights and adjusted heights^†^ on year of birth in the total samples of Hindu males 18–84 years and Muslim males 18–76 years of age born between the 1891 and 1957, and among subsamples of each group born in 1891 through 1930 and in 1930 through 1957; the regression coefficients provide an estimate of change in each variable over time (i.e., year of birth).

	Regression coefficients					Regression coefficients				
	r	β± SE_b_ (cm/yr)	95% CI		F	r	β± SE_b_ (cm/yr)	95% CI		F
	Hindu					Muslim				
A	Total sample (n = 38,776) born 1891–1957					Total sample (n = 5139) born 1899–1957				
Height	0.025	0.015 ± 0.003	0.009	0.020	24.68*	0.040	0.022 ± 0.008	0.007	0.037	8.19**
Adjusted height	− 0.034	− 0.020 ± 0.003	− 0.026	− 0.014	45.62*	− 0.024	− 0.013 ± 0.008	− 0.028	0.002	3.02
B	Sample (n = 17,685) born 1891–1930					Sample (n = 2219) born 1899–1930^#^				
Height	0.035	0.039 ± 0.008	0.023	0.056	21.79*	0.047	0.054 ± 0.026	0.003	0.105	4.31***
Adjusted height	− 0.042	− 0.047 ± 0.008	− 0.064	− 0.031	31.37*	− 0.041	− 0.046 ± 0.026	− 0.098	0.005	3.17
C	Sample (n = 31,910) born 1930–1957					Sample (4317) born 1930–1957				
Height	0.008	0.010 ± 0.007	− 0.003	0.024	2.25	0.026	0.033 ± 0.019	− 0.005	0.071	2.98
Adjusted height	− 0.009	− 0.010 ± 0.007	− 0.024	0.003	2.32	0.009	0.011 ± 0.019	− 0.027	0.049	0.34

*p < 0.001; **p < 0.01; ***p < 0.05.

^†^The samples for adjusted heights include the measured heights of individuals 18 through 34 years and adjusted heights of individuals 35 + years (i.e., measured heights plus the estimated height loss with age, see text for details).

^**#**^The group of Muslims born in the second period (B) encompassed men aged 18–76 (born in the years 1899–1930).

Across the interval of birth years for the total samples of Hindu (1891–1957) and Muslim men (1899–1957), the regression coefficients for measured heights suggest a small but significant secular increase in height among Hindu (0.015 ± 0.003 cm/year, p < 0.001) and Muslim (0.022 ± 0.008 cm/year, p < 0.01) men (Table 3A). However, the regressions for the combined samples of men 18–34 years (measured heights) and of men 35 + years (adjusted heights) indicate a small secular decline in height among both Hindu (-0.020 ± 0.003 cm/year, p < 0.001) and Muslim (-0.013 ± 0.008 cm/year, not significant) men.

Among men born in the 1890s through 1930 (a major interval of the British Raj), the regressions for measured heights of indicate larger secular increases in both Hindu (0.039 ± 0.008 cm/year, p < 0.001) and Muslim (0.054 ± 0.026 cm/year, p < 0.05) men. When heights of men 35 + years are adjusted for estimated stature loss with age, regressions in the two groups suggest similar secular declines in Hindu men, − 0.047 ± 0.008 cm/year (p < 0.001) and in Muslim men, − 0.046 ± 0.026 cm/year (not significant) (Table 3B).

Regressions of the heights of men born in the 1930s through 1957 (the closing years of British rule, famine, World War II, early years of independence), indicate negligible secular change in measured heights of Hindu men, 0.010 ± 0.007 cm/year and a secular gain among Muslim men, 0.033 ± 0.019 cm/year (Table 3C). When the heights of men 35–44 years in the sample were adjusted for estimated stature loss with age, the estimated secular change is negligible and negative among both Hindu men, − 0.010 ± 0.007 cm/year, and negligible but positive among Muslim men, 0.011 ± 0.019 cm/year.

When the results of the regressions are expressed per decade, estimated secular changes in measured heights in the total samples of Hindu and Muslim men are small and reasonably similar, 0.15 cm/decade and 0.22 cm/decade, respectively. When heights of men 35 + years were adjusted for estimated height loss with age, the corresponding estimates of secular changes are also similar but negative, − 0.20 cm/decade among Hindu and − 0.24 cm/decade among Muslim men.

Among men born in the 1890s through 1930, the interval of the British Raj, estimated secular change in measured heights is slightly larger among Muslim (0.54 cm/decade) than Hindu (0.39 cm/decade) men. Corresponding estimates of secular change when heights of men 35 + years were adjusted for estimated height loss with age are virtually identical but negative in both Hindu (− 0.47 cm/decade) and Muslim (− 0.46 cm/decade) men.

Among men born during the closing years of the British Raj which also included several famines and World War II, and the early years of independence (1930 through 1957), estimated secular changes in measured heights are slightly larger among Muslim (0.33 cm/decade) than Hindu (0.10 cm/decade) men. After adjusting heights of men 35–44 years for estimated height loss with age, the estimates indicate negligible secular changes in both Muslim (0.11 cm/decade) and Hindu (− 0.11 cm/decade) men.

## Discussion

Hinduism and Islam are the two largest religions in India, although Hindus represented 80% and Muslims only 14% of the Indian population in the 2011 census^12^. It may be potentially misleading if Animists (tribes) and Atheists are not counted separately, although both are generally included as Hindus. Nevertheless, in a country of more than 1.4 billion people, the Census is the only authentic source of population composition. Hindus are the significant majority in all Indian states except Jammu and Kashmir and in Punjab, where Muslims predominate^14^. It should be noted, however, that Hindus and Muslims are not homogenous populations. The two religious groups are divided into castes, sects, and cultural groups. Scheduled Castes (SCs), Scheduled Tribes (STs) and a large number of socially Backward Castes (BCs) comprise the Hindu community, while the Sunnits, Shias, Bohras and others are part of the Muslim community^14^. With the independence of India in 1947, a large number of Muslims moved to Pakistan. In the first census shortly after independence in 1951, 35 million Muslims in India constituted the largest minority in the country, while the Hindus represented 304 million^14^.

Hindus and Muslims differ in lifestyle, traditions, and customs, which influence attitudes regarding family, community, reproduction and also nutritional and perhaps lifestyle habits, among other considerations. Differences between Hindus and Muslims are also apparent in fertility. In urban and rural regions of Indian states in 1981, total fertility rates and general marital fertility rates were higher among Muslims than Hindus^14^, and this pattern continued into the present^14,20^. Child and adult mortality was also significantly lower in Muslim than Hindu communities^14^ and this pattern persisted into the twenty-first century^14,21^. Estimated mortality at 70 years and the probability of death between 15 and 60 years was higher, on average, among Hindus than Muslims^21^. And in the context of the present study, the trends in height, weight and the BMI among Muslim and Hindu men also favored the former.

In contrast and perhaps somewhat unexpectedly, economic indicators in India have favored Hindus^22^. Both during the British Raj and after independence, the economic position of the Hindus was better than that of the Muslims in India. Muslims were also under-represented in the most dynamic sectors of the Indian economy during the British Raj. For example, their role in the production and transport of cotton in the western regions of the country, the two most dynamically developing sectors, was negligible. On the other hand, Muslims were owners of tea plantations and processing enterprises in the eastern regions. Shortly before independence, Muslims were owners of only two jute mills and were underrepresented in industry and other enterprises in the northern region. It has been suggested that the reasons for this discrepancy were cultural. In the Islamic system of property inheritance, the principles of partnership and Islamic trusts (called waqfs) limited the participation of Muslims in large enterprises and long-term projects. These cultural practices and institutions likely limited opportunities for Muslims in the economy^23^. According to 2011 Census of India, about 40% of Muslims lived in urban areas compared to 29% of Hindus^12^. In a comparison of socio-economic and demographic factors associated with land ownership, literacy, educational attainment, employment, and consumption expenditure among different religious groups in India, including Hindus and Muslims among others, Muslims fared worse than Hindus (including the lowest castes, Scheduled Caste, and Scheduled Tribes) in all variables considered^14^. And in 1987–1988, about 44% of Hindus from rural areas were employed in agriculture, 28% in agricultural labour and only 11.7% in non-agricultural sector; the corresponding estimates for Muslims were 36%, 24% and 21%, respectively^14^. Also in the 1980s, about 47% of Hindus were employed in regular waged/salaried occupations compared to only 29% among Muslims. On the other hand, 53% of Muslims were self-employed compared to only 36% of Hindus^14^. Somewhat surprisingly, more Muslims than Hindus were overrepresented among the landless in India^14^. Muslims also lag behind Hindus in education levels and the inequalities in education affect the quality of employment among Muslims, whose percentage in the labor market is much lower than that of Hindus^22^. Participation in the labour force is also lower among Muslim compared to Hindu women, 13.3% and 21.2%, respectively. Access to health care among mothers and children is also poorer among Muslims^22,24–28^.

Despite the seemingly adverse socio-economic status and the position of Muslims compared to Hindus in Indian society, Muslim men in the present study were, on average, taller and heavier than Hindu men; also sitting height and the sitting height/height ratio were significantly greater among Muslim men (Fig. 2A and B). In both Muslims and Hindus, estimated leg length (estimated as height minus sitting height) accounted for about 50% of the secular increase in height, i.e., estimated leg length and sitting height contributed equally to the secular increase in the height of the men (Table 1). Socio-economic adversity in childhood is associated with delayed early growth, shorter adult stature and leg length; the latter is the component of height that is most sensitive to environmental conditions early in postnatal life^29^. Leg length appears to be a particularly sensitive indicator of childhood socio-economic circumstances, and a greater part of the difference in stature between socio-economic groups was caused by differences in leg length rather than trunk length^30^. Research has also emphasized that leg length was a component of stature most strongly associated with childhood diet and socio-economic status^30^. In the present study, estimated leg length does not systematically differ between Muslim and Hindu men. By inference, it is possible that marginal or poor nutritional conditions during the British Raj, which overlapped fetal development and early childhood of both Hindu and Muslim men, may have negatively affected both groups to the same extent. In both Muslims and Hindus, however, estimated leg length increases slightly between 18 and 29 years, while sitting height declines with age. It is possible that the slight increase in height in the youngest birth cohorts was associated with an increase in leg length. Muslim men also have, on average, a greater BMI than Hindu men at most ages. While height and estimated leg length reflect the interaction of genotype and environmental conditions (health, diet, family socioeconomic status, living conditions, among others) during the course of growth and maturation, the BMI is largely an indicator of the balance between energy intake and energy expenditure^31^.

The greater heights in Muslims than Hindus have also been confirmed in studies of contemporary Indian populations. Based on data from the Indian National Family Health Survey for 2005–2006, for example, Muslim women in each wealth quintile group were taller, on average, than Hindu women^32^. Research on children 5 years of age also shows that Hindus have a higher likelihood of wasting than Muslims, 16% and 6%, respectively. The Muslim advantage in indicators of undernutrition compared to high-caste and low-caste Hindus may have been influenced by certain ‘unobserved’ behavioral and cultural differences^22^. Divisions in a society may also play an important role in the stratification of body size by religion. For example, men from the poorest Hindu castes: Scheduled Tribe, Scheduled Castes and Backwards Castes had the shortest heights (< 152 cm), 5.4%, 3.6% and 3.4%, respectively, while significant numbers were also classified as "short" (153–162.9 cm), 54.7%, 48.7% and 44.8%, respectively^33^. In contrast, Hindus in General Castes (the highest on the Hindu social ladder), and Muslims in the sample comprised on 1–2% of the shortest height sample, while 34.1% of the Hindus in General Castes and 38.4% of Muslims were classified as short^33^. Differences in adult stature are generally viewed as reflecting differences in social and economic position reflected in occupation and income, and in turn conditions of living among social/ ethnic groups which influence nutrition and health care during infancy, childhood, and adolescence^33^.

Trends in the present study also suggest that an important factor in the size differences between Muslim and Hindu men may be related to quality of diet, specifically prescribed vegetarianism among Hindus^13^. Over 83% of Hindus indicate that they are either vegetarian or have restrictions on the kinds of meat they can consume, although some do in fact occasionally eat meat, fish, and eggs (note that beef and pork are not permitted and have a religious taboo)^34^. The vegetarian diet is associated with a low intake of saturated fats and cholesterol and a high intake of dietary fibre and many health-promoting phytochemicals. The latter may influence weight status and contribute to lower cholesterol and/ or blood pressure^34^. In a national survey of adult non-pregnant women, the prevalence of underweight was 24% among Hindus compared to 21% among Muslims, and the prevalence of anaemia was 53% among Hindus and 50% among Muslims^35^. In the present study, 52% of the Hindu men surveyed in the 1970s had a BMI < 18.5 kg m^2^, compared to 46% of Muslim men (Table 2). A study of the diets of religious communities in Gujarat noted that Hindu Brahmins were at greater risk of anaemia than meat-eating Muslims^36^. On the other hand, overweight and obesity were significantly lower among Hindus than among Muslims, 17% and 22%, respectively^35^. However, the prevalence of overweight and obesity among the Hindu and Muslim men surveyed in the 1970s was quite low and did not differ between groups (Table 2). Vegetarian diets of children and adults are deficient in vitamin B12, iron, zinc, selenium, and omega-3 fatty acids^37^, while vitamin B12 deficiency was noted in 51% of pregnant Indian women and 44% of their infants at 6 weeks of age^38^.

Unfortunately, information on the nutritional status of Hindu and Muslim populations during the interval spanning the birth years of the men in the present study, i.e., turn of the century through the 1970s, is lacking. Given the economic and demographic stagnation described in the previous studies of the Indian men^18,19^, it is reasonable to assume that present-day dietary differences between Hindus and Muslims were also apparent in the mid-nineteenth century through the 1970s. It is also likely that food shortages during the interval of the British Raj affected both Hindu and Muslim men. Of relevance, results of the regressions of the heights on year of birth (Table 3) among men born in the 1930s through 1957 (closing years of British rule, famine, World War II, and early years of independence), indicated negligible secular change in the heights of Hindu and Muslim men, which was consistent with the social and economic stagnation during this interval.

Studies of contemporary samples indicate that overweight and obesity predominate among Muslim (22.4% and 7.4%, respectively) compared to Hindu (19.3% and 6.3%; respectively) women. In contrast, Hindu women have a higher prevalence of underweight (14.9%) than Muslim (9.8%) women, while the prevalence of normal weight status is slightly higher among Muslim (60.3%) than Hindu (59.5%) women^39^. The greater heights and weights among Muslims may be related to higher birth weights. Although differences between Muslims and Hindus are relatively small: normal and heavy birth weights are more prevalent among Muslims (80.8% and 3.4%) than among Hindus (79.6% and 2.7%), while low birth weight is more prevalent among Hindus (17.7%) than Muslims (15.8%)^39^. The differences, though relatively small, may reflect aspects of maternal health that are not directly related to maternal SES^40^. A factor that may influence the advantage of the biological conditions of Muslims over Hindus is a lower degree of preference for sons among Muslim compared to Hindu families. It is possible that Muslim women receive an equal share of household resources during childhood, which may have an influence on height and weight as adults which may influence the birth weight of offspring^39,41^. Research has also confirmed a lesser gender discrimination in the distribution of resources within Muslim than in Hindu households^42,43^. It has also been suggested that other advantages among some Muslims may be associated with closer kinship (psychological and social support), better health of Muslim mothers due to lack of sex discrimination, and lower propensity to work outside the home^42^. Socioeconomic status among Muslims may also not have been as important a determinant of health or biological status as, for example, attitudes and relations at the individual or community level^42,44,45^. The strengths of Muslim familial and kin relationships play a very important role in child-oriented health measures. The close kinship networks and marital circles in Muslims may have contributed to the feeling of greater security and therefore, greater social and psychological well-being; a related factor is likely the “tight-knit circles” related to marriage practices in Muslim families^46^. This pattern of relationships likely provided for Muslim women and future mothers strong family support associated with childcare which may have provided the basis for positive long-term effects on the offspring which persisted into adulthood.

In summary, Muslim men were, on average, taller and heavier than Hindu men, although the literature suggests generally poorer living and economic conditions for Muslims than Hindus during the British Raj and after independence. This would seem to suggest that social determinants within the respective communities identified by religion play an important role, for example, sex selection or lack thereof, family and community ties, social norms and networks, food preferences, etc., and not socioeconomic status per se contributed to the advantage in height and weight among Muslim compared to Hindu men. On the other hand, conditions in India during the interval of the British Raj and after independence were not sufficient to support positive secular change in the heights of both Muslim and Hindu men.

## Material and methods

### Ethics statement

Formal human subject review boards came into existence in India with the National Research Act of 1974, which post-dates the first survey wave of the Anthropological Survey of India. However, the first Anthropological Survey had its own internal review board which focused on the protection of human rights and all methods were carried out in accordance with its relevant guidelines and regulations. The research was approved by the Anthropological Survey of India Ethics Committee directed by Dr B. S. Guha. Moreover, all experimental protocols were approved by the internal review board of the first Anthropological Survey (the Anthropological Survey of India Ethics Committee). As required by the internal board, all participants were informed of the survey objectives and provided verbal informed consent to participate in the survey. The raw data from the Survey were published in a series of volumes for the respective states^47–49^. The survey data were used previously to evaluate nutritional and health status differences among tribes and castes, and by socioeconomic status and geographic regions^50,51^.

### Participants and methods

The data are from the Anthropological Survey of India in the1970s. The survey was limited to men due to the lack of female researchers, in addition to the conservative societal conditions. In rural areas, for example, men were not permitted to measure women. A related factor was the high rate of illiteracy among females, especially in rural areas. The participants in the 1970s surveys were described as healthy and active; the surveys also included special efforts to exclude closely related individuals, i.e., brothers and fathers and sons, and also individuals with any type of physical deformity. The surveys included age, several anthropometric dimensions, geographic region of residence and religion (Hindu, Muslim) for each participant.

India had a population of 548 million in 1970^52^ and of 681 million in 1980^20^. The states and territories represented in the surveys accounted for 61% of the total Indian population in 1970. The present analysis is limited to 43,879 males 18 to 84 years of age among whom height, weight and sitting height were measured by trained physical anthropologists using calibrated weighing scales and anthropometers for height and sitting height and following standard techniques^53^. Sitting height was subtracted from height to provide an estimate of leg length. The ratio of sitting height to height (sitting height [cm]/height [cm] × 100), and the BMI (weight [kg]/height [m^2^]) were calculated. Based on the BMI, the weight status of each individual was classified relative to criteria of the World Health Organization criteria^54,55^ and to criteria proposed for Asia–Pacific populations^56^.

A decline in height with increasing age among adults is well-documented, though estimated declines vary among samples^57,58^. Cumulative height loss with age among men 35 years and older was thus estimated with the equation of Sorkin and colleagues^59,60^:

$${\text{Height loss }}\left( {{\text{cm}}} \right) \, = \, \left( { - 0.00{21 }*{\text{ age}}^{{2}} } \right) \, + \, \left( {0.{1258 }*{\text{ age}}} \right) \, - { 1}.{8829}$$

The equation was based on 16 samples of men of European ancestry (Europe, 8; United States, 7, Australia, 1) who were observed on at least two occasions spanning several years. Estimates of height loss with age in longitudinal samples of Indian men are apparently not available. The estimated height loss was added to the current height of each individual 35 + years of age to provide an estimate of his maximum height (labelled adjusted height).

The youngest age group included subjects 18 through 24 years (birth years 1951–1957), while the oldest group included subjects ≥ 60 years (60–84 years, birth years 1891–1915). The other age groups spanned five-year intervals, 25–29 years, 30–34 years … through 55–59 years. Descriptive statistics (means and standard deviations) for age, year of birth, height, weight, BMI, sitting height, estimated leg length and the sitting height/height ratio were calculated by age groups. Means and standard deviations were also calculated for adjusted heights, i.e., heights adjusted for estimated height loss with age, among men 35 + years of age. Age group specific multiple analyses of covariance (MANCOVA), with age, age squared and geographic region as covariates, were used to evaluate the differences in height, adjusted height, weight, the BMI, sitting height, estimated leg length, and the sitting height/height ratio between the Hindu and Muslim men. The prevalence of men by weight status based on WHO criteria for the BMI was also calculated by age groups.

Three pairs of separate linear regressions were used to evaluate the influence of year of birth on heights of the Hindu and Muslim men. The initial regressions were performed for the respective total samples of Hindu and Muslim men (A), and then for subsamples born in two intervals, 1891–1930 (B) and 1930–1957 (C). The samples of Hindu and Muslim men partitioned by year of birth considered two important events in the history of India during birth year interval of the participants^61–63^. The first group included men born in 1891 through the 1930s and reared during the interval of the British Raj in India (1858 through August 1947). The second group included men born in the 1930s through 1957 and reared during the closing years of the British Raj, including overt struggles for independence (the 1930s–1947), World War II (1939–1945), the Bengal famine (1940–1943) and other famines, and the transition to the independent state of India (late 1947 onward). For each birth interval regressions were done for measured heights in the respective samples of Hindus and Muslims, and for the combined total samples which included measured heights of men < 35 years and adjusted heights of men 35 + years. The regression coefficients provide an estimate of the change in height over time (years of birth).

All statistics were carried out with the Statistical Package for the Social Sciences (IBM SPSS Statistics 19, 2011). Significance was set at p < 0.05.

## Data Availability

The datasets used and/or analysed during the current study are available from the corresponding author on reasonable request.
